# T Cell Receptor Expression Timing and Signal Strength in the Functional Differentiation of Invariant Natural Killer T Cells

**DOI:** 10.3389/fimmu.2019.00841

**Published:** 2019-04-26

**Authors:** Nyambayar Dashtsoodol, Sabrina Bortoluzzi, Marc Schmidt-Supprian

**Affiliations:** ^1^Department of Hematology and Medical Oncology, Klinikum rechts der Isar and TranslaTUM Cancer Center, Technische Universität München, München, Germany; ^2^Department of Microbiology and Immunology, School of Biomedicine, Mongolian National University of Medical Sciences, Ulaanbaatar, Mongolia

**Keywords:** NKT, CD1d, lymphocyte, development, functional subset, thymus, T cell receptor, developmental pathway

## Abstract

The CD1d-restricted Vα14 invariant NKT (iNKT) cell lineage in mice (Vα24 in humans) represents an evolutionary conserved innate-like immune cell type that recognizes glycolipid antigens. Because of their unique ability to promptly secrete copious amounts of both pro-inflammatory and anti-inflammatory cytokines, typically produced by different T helper cell types, iNKT cells are implicated in the regulation of various pathologic conditions such as infection, allergy, autoimmune disease, maintenance of transplantation tolerance, and cancer. This striking multifaceted role in immune regulation is correlated with the presence of multiple functionally distinct iNKT cell subsets that can be distinguished based on the expression of characteristic surface markers and transcription factors. However, to date it, remains largely unresolved how this puzzling diversity of iNKT cell functional subsets emerges and what factors dictate the type of effector cell differentiation during the thymic differentiation considering the mono-specific nature of their T cell receptor (TCR) and their selecting molecule CD1d. Here, we summarize recent findings focusing on the role of TCR-mediated signaling and discuss possible mechanisms that may influence the sub-lineage choice of iNKT cells.

## Introduction

Innate-like T lymphocytes are a special group of immune cells assumed to play important immunoregulatory functions by linking and orchestrating the functions of multiple cell types of the innate and adaptive arms of the immune system (1, 2).

The invariant NKT (iNKT) cells, often referred to as Vα14 iNKT cells in mice (Vα24 in humans), represent an evolutionary conserved innate-like immune cell type characterized by the expression of a unique semi-invariant T cell receptor (TCR). This TCR is composed of a single invariant α-chain Vα14Jα18 (encoded by *Trav11Traj18*) usually paired with a limited range of TCRβ-chains, mostly Vβ8.2, Vβ7, or Vβ2 (TRBV13-2, TRBV29, and TRBV1), in mice and a Vα24Jα18 TCRα-chain (encoded by *Trav10Traj18*) paired exclusively with Vβ11 (TRBV25) in humans. As opposed to the conventional peptide-recognizing CD8 T cells or CD4 T cells, iNKT cells are specialized in recognizing glycolipid antigens including alpha-galactosylceramide (α-GalCer) presented by the monomorphic MHC class I-like CD1d molecule (3–7). Owing to the early availability of specific analysis tools such as CD1d tetramers (8, 9), as well as various gene-manipulated mouse models that either lack (10–17) or overexpress (18–22) these cells, to date iNKT cells represent the best-studied innate-like T lymphocyte lineage.

The hallmark feature of iNKT cells is their unique ability to secrete very large amounts of both pro-inflammatory and anti-inflammatory cytokines, typically produced by T helper cell type 1 (T_H_1), T_H_2, and T_H_17 cells. Cytokine secretion occurs rapidly upon activation and does not require clonal expansion or antigenic priming. Therefore, in line with their innate-like ability to robustly produce multiple immunoregulatory cytokines, iNKT cells were implicated in the regulation of various pathologic conditions such as infection, allergy, autoimmune disease, maintenance of transplantation tolerance, and cancer (23, 24). Because of this unique feature to elicit protective, regulatory, and pathogenic functions, it was proposed that iNKT cells constitute a heterogenous population. In fact, recent publications have demonstrated the presence of multiple functionally distinct subsets with discrete cytokine polarization within the iNKT cell lineage that can be distinguished based on the expression of characteristic surface markers and transcription factors (Table 1) (25, 26). The differentiation of iNKT cells proceeds within highly restricted context dictated by the recognition of self-glycolipids on CD1d by their semi-invariant TCR. To date, it remains largely unresolved how this puzzling diversity of iNKT cell functional subsets emerges and what factors dictate the type of effector cell differentiation during thymic differentiation.

**Table 1:** Differential expression of surface markers and transcription factors on iNKT cell functional subsets.

**iNKT subsets**	**Key secreted cytokines**	**Signature transcription factors**	**Surface marker**	**References**
iNKT1	• IFN-γ • TNF-α	• T-bet^+^ • PLZF^low^	• CD122^+^ • CXCR3^+^ • IL-17RB^−^ • CD49a^+^ • CD43HG^−^ • ICOS^−^ • CD27^+^	(25–28)
iNKT2	• IL-4 • IL-5 • IL-13	• PLZF^high^ • GATA-3^high^	• IL-17RB^+^ • CD4^+^ • ICOS^+^ • CD43HG^inter^ • CD27^+^	(25–28)
iNKT17	• IL-17 • IL-22	• RORγt^+^ • PLZF^inter^	• ICOS^+^ • CCR6^+^ • CD103^+^ • NRP1^+^ • CD4^−^ • IL-17RB^+^ • CD43HG^+^ • CD138 • IL-23R • CD27^−^	(25–29)
iNKT10	• IL-2 • IL-10	• E4BP4^+^ • PLZF^−^	• PD1^+^ • CD49d^+^ • NRP1^+^ • CD4^+^	(27)
iNKT_FH_	• IL-21	• BCL-6^+^	• PD1^+^ • CXCR5^+^ • CD4^+^	(27)

*iNKT, invariant NKT*.

As a number of excellent reviews have extensively covered advances in the iNKT cell developmental field (27, 30–34), in this mini-review, we attempted to summarize recent findings focusing on the role of TCR-mediated signaling and discuss possible mechanisms that may influence the sub-lineage choice of iNKT cells. A better understanding of the mechanisms underlying the differentiation of iNKT cell functional subsets will eventually help in designing new strategies to explore the therapeutic potential of these cells for the benefit of immunocompromised patients.

## iNKT Functional Subsets

In contrast to conventional T cells, iNKT cells can acquire functional maturity in the thymus before their egress to peripheral tissues. Historically, a linear differentiation model was proposed, which postulated that CD1d-selected CD24^+^ stage 0 (st0) immature iNKT cells mature through sequential stages: via CD44^low^ NK1.1^−^ stage 1 (st1) iNKT cells characterized by IL-4-producing capabilities to CD44^high^ NK1.1^−^ (st2) iNKT cells with IL-4- and IL-17-biased features, and finally to terminally matured CD44^high^ NK1.1^+^ (st3) iNKT cells with IFN-γ-biased polarization (35). However, direct evidence of differentiation of st1 and st2 iNKT cells into terminally matured st3 iNKT cells was not demonstrated (36). The first evidence probing the above question on whether IL-4-producing thymic iNKT cells can give rise to IFN-γ-producing iNKT cells was addressed by Watarai et al., who demonstrated that IL-4-producing IL-17 receptor B (IL-17RB) expressing thymic iNKT cells do not generate IFN-γ-producing T-bet^+^ thymic iNKT cells upon intrathymic transfer (25).

More recently, based on intracellular staining patterns of lineage-specific transcription factors such as T-bet, GATA-3, PLZF, and RORγt, Lee et al. demonstrated that there are at least three distinct iNKT functional subsets in the thymus, designated as iNKT1, iNKT2, and iNKT17, akin to the classification of classical CD4 T helper cell types (T_H_1, T_H_2, and T_H_17 cells, respectively) (26). iNKT1 cells express NK cell-related markers, are T-bet^+^ PLZF^low^, and produce mainly IFN-γ. Moreover, iNKT2 cells are GATA-3^high^ PLZF^high^ and produce high levels of IL-4, whereas iNKT17 cells are defined as RORγt^+^ PLZF^intermediate^ and produce IL-17 upon stimulation. Thus, NKT functional subsets can be distinguished on the basis of their expression profile of characteristic surface markers and signature transcription factors (Table 1).

iNKT cells also can be subdivided into CD4^+^ and CD4^−^ subsets, where both murine and human CD4^−^ subsets show T_H_1-biased cytokine polarization and enhanced cytotoxic activity compared with CD4^+^ counterparts (37–39). In line with this, granzyme A (Gzma) and granzyme B (Gzmb), key genes involved in NK-triggered killing, are expressed specifically on CD4^−^ iNKT cells (40). Of note, hepatic CD4^−^ iNKT cells possess superior antitumor activity compared with thymic or splenic CD4^+^ and CD4^−^ iNKT cells, suggesting that organ-specific mechanisms might dictate the functional capabilities of resident NKT cells (39). Although it remains incompletely understood whether the development of these diverse functional NKT cell subsets is related to the existence of dedicated precursor cells or is due to specific differentiation programs, a revised model, termed “lineage diversification,” is proposed, which suggests thymic iNKT functional subsets represent stable distinct lineages rather than functional maturation stages (26, 30).

In addition to these subsets, iNKT follicular helper cells (iNKT_FH_) and IL-10-producing iNKT10 cell subsets were described (41–44). Although it is unclear whether iNKT_FH_ cells originate from the thymus, iNKT10 cells can arise in the thymus of F108Y mice, which harbor a mutation in the TCRβ-chain that results in altered iNKT TCR conformation (45). In addition to this, iNKT cells convert into IL-10-producing cells upon repeated injection with agonistic glycolipid α-GalCer (46), which suggests iNKT cells might have some degree of sub-lineage plasticity. In regard to this, there is a report on induction of TGF-β-dependent FOXP3^+^ iNKT cells in the periphery upon administration of α-GalCer (47).

Moreover, the relative frequencies of functional subsets differ between inbred mouse strains, such as C57BL/6 mice possess mostly iNKT1 cells, while BALB/c mice have a significantly larger iNKT2 cell subset. Additionally, iNKT cells are tissue resident and show unique tissue distribution patterns (27, 48).

## Thymic Selection and Differentiation

**Figure 1:**
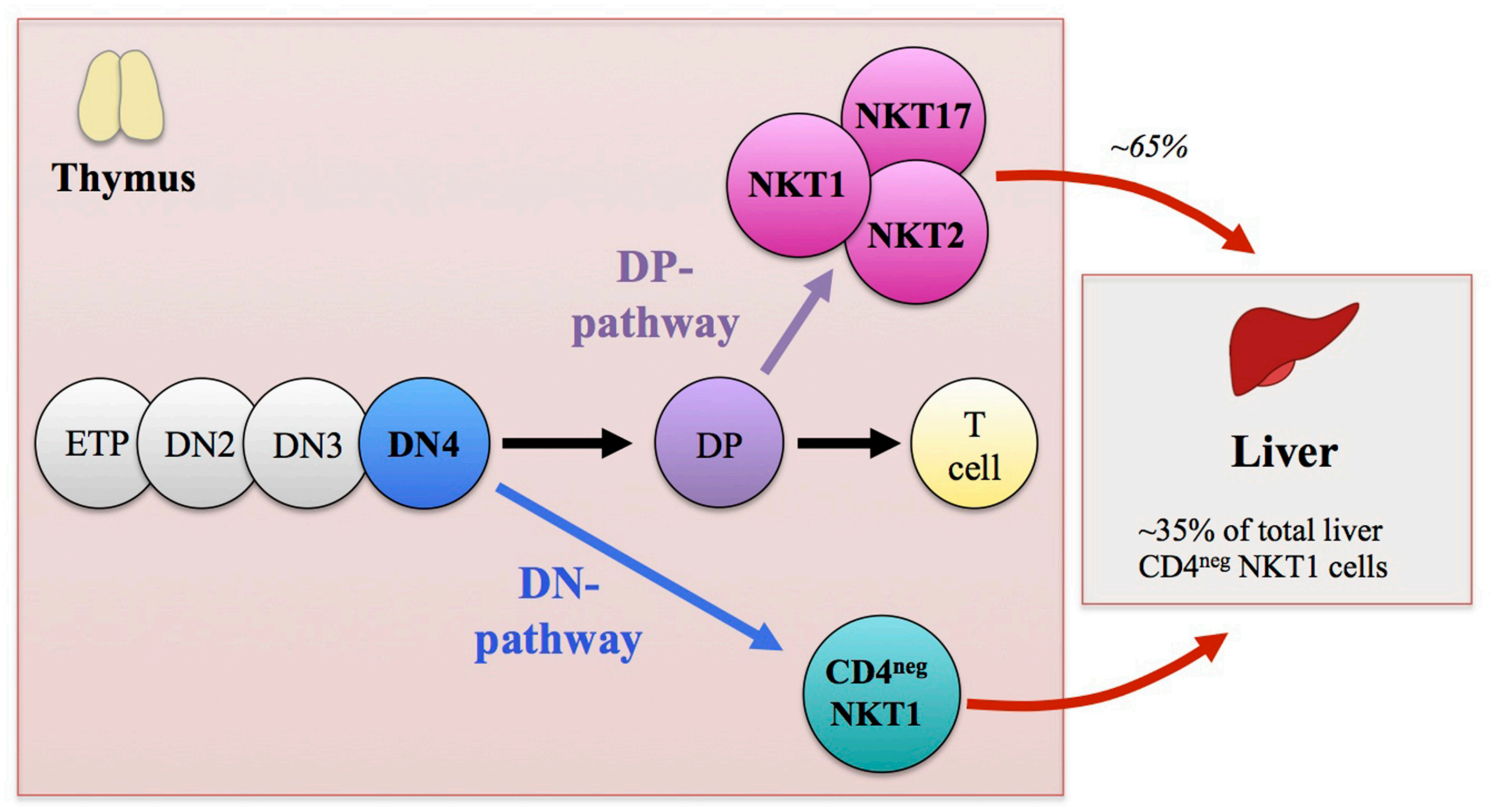
Schematic representation illustrating the thymic development of invariant NKT (iNKT) cells. Early thymic development of iNKT cells mirrors that of αβ T lymphocytes. Thymocyte precursor cells differentiate through CD4^−^CD8^−^ double-negative (DN)1-to-DN4 stages to become CD4^+^CD8^+^ double-positive (DP) thymocytes. In contrast to peptide-recognizing T cells that are selected at the DP stage of thymic ontogeny by highly polymorphic MHC class I or II molecules expressed on thymic radio-resistant stromal cells, iNKT cells are selected with glycolipid antigen-presenting monomorphic CD1d-expressing thymocytes. The majority of iNKT cells (including iNKT1, iNKT2, and iNKT17 functional subsets) derive from DP thymocyte precursors that express the invariant Vα14Jα18 TCRα-chain paired with a Vβ8/7/2 TCRβ-chain. These DP precursors commit to the iNKT cell lineage after being positively selected by CD1d-expressing cortical thymocytes. However, a fraction of CD4^−^ iNKT cells develops directly from the DN4 stage thymocyte precursors without passing through the DP stage of thymic ontogeny. Although both the DN and DP pathways contribute to the generation of CD4^−^ iNKT cells, the DN pathway preferentially gives rise to IFN-γ-producing iNKT1 cells with augmented cytotoxic properties. In addition, the DN pathway generates CD4^−^ iNKT cells with distinct peripheral distribution patterns compared with DP pathway-generated counterparts. The CD4^−^ iNKT cells generated by the DN pathway are found mainly in the liver, where DN thymocyte-origin CD4^−^ iNKT cells constitute ~35% of the total CD4^−^ iNKT cells, while ~65% of the total CD4^−^ iNKT cells are generated through the DP pathway.

The development of the iNKT cell lineage proceeds in the thymus, where the precursor cells that successfully assembled their antigen receptor through recombination mediated by the recombination-activating gene (RAG) are positively selected on CD1d-expressing cortical CD4^+^CD8^+^ double-positive (DP) thymocytes (49). Although the majority of iNKT cells are generated from DP thymocytes, it was recently demonstrated that a fraction of iNKT cells develops directly from the CD4^−^CD8^−^ double-negative (DN) stage of thymic ontogeny, bypassing the DP stage. Fate-mapping experiments as well as conditional ablation of RAG2 at the DP stage demonstrated that a fraction of CD4^−^ iNKT cells were able to develop directly from DN-stage thymocytes without passing through the DP stage (50). This suggested a scenario in which DN-stage thymocyte precursors expressing rearranged Vα14Jα18 generated by random rearrangement events are positively selected on CD1d and develop into mature CD4^−^ iNKT cells (Figure 1).

Although the exact nature of endogenous ligands presented by CD1d responsible for positive selection of iNKT cells remains to be determined, it was recently reported that mammalian α-linked glycosylceramides represent a candidate for an iNKT cell self-ligand in mice (6). In striking contrast to conventional T cells that undergo clonal deletion upon receiving strong signaling via TCR-MHC interaction, iNKT cells are considered to be selected via strong TCR signals in the thymus. The evidence suggesting agonist selection of iNKT cells is based, among others, on the previously activated and/or memory phenotype of iNKT cells as well as the elevated expression levels of markers associated with TCR signaling such as Egr2 (51) and Nur77 (52) on early iNKT cells. In addition to this strong TCR signaling during selection, the development of iNKT cells requires homotypic interactions between signaling lymphocytic activation molecule family (SLAM) receptors Slamf1 (CD150) and Slamf6 (Ly108) expressed on selector DP thymocytes (53). Reporter mice for TCR signal strength revealed that iNKT cells receive TCR signals during a relatively short developmental time window exclusively at the st0 immature iNKT cell stage (52). These selection signals induce Ras- and Ca^2+^-dependent transcription factors Egr1 and Egr2, where Egr2 is shown to directly regulate the expression of promyelocytic leukemia zinc finger (PLZF) (51). PLZF represents a key regulator of the innate-like effector functions of iNKT cells (54, 55). However, there is currently no clear consensus on how PLZF is induced, as it was shown that TCR stimulation is not sufficient to induce PLZF expression on pre-selection DP thymocytes (56), suggesting other unknown signals are likely to be required for the development of PLZF-expressing innate T cells such as iNKT. One important factor might be the property of the positively selecting cell type, as CD4 T cells selected by MHC class II-expressing thymocytes express PLZF and show innate-like characteristics (57–59). Moreover, as PLZF expression can be detected in a subset of DN2 thymocytes (60), it is possible that those early-stage precursors expressing PLZF could differentiate later into a PLZF-expressing iNKT cells. In addition to this, premature expression of PLZF on DP thymocytes was demonstrated in pTα/Id2/Id3-deficient mice (61), which suggests a heterogeneity within pre-selection developmental intermediates (62, 63).

Much less is known regarding the negative selection of NKT cells. It was reported that intrathymic injection of α-GalCer and forced expression of CD1d on thymocytes or thymic antigen-presenting cells (APCs) such as dendritic cells (DCs) led to reduced numbers of iNKT cells (64–66). Whether negative selection shapes the iNKT cell population in normal development remains unclear.

## Developmental Timing of T Cell Receptor Expression

The early thymic development of iNKT cells presumably mirrors that of conventional αβ T lymphocytes, where the early multipotential bone marrow-derived progenitors differentiate through tightly regulated DN stages 1 to 4 defined based on expression of CD117, CD25, and CD44 markers (67, 68). Major commitment to the αβ T cell lineage occurs at the DN3 stage, where rearrangement of the TCRβ-chain genes and subsequent beta-selection take place (69). Those DN3-stage thymocytes expressing functional pre-TCR, composed of pre-TCRα/TCRβ, differentiate further into the DN4 stage to become DP thymocytes. Although it is well-accepted that rearrangement of the TCRα-chain occurs at the DP stage, there are some reports demonstrating the presence of TCRα transcripts within DN-stage thymocytes prior to differentiation into the DP stage (70–72). In this regard, it was reported that the DN4-stage thymocytes express Vα14Jα18 iTCR and RAG transcripts and possess iNKT cell potential *in vivo* (72). Furthermore, TCR sequencing experiments revealed the presence of out-of-frame *Trav11Traj18* sequences, providing compelling evidence for ongoing stochastic TCRα-chain rearrangements within late DN-stage thymocytes (50).

It seems that iNKT TCR expression during the late DN stage of thymic ontogeny plays a role in shaping the iNKT functional subset choice. Although both DN and DP pathways contribute to the generation of CD4^−^ iNKT cells, the former pathway “preferentially” gives rise to IFN-γ-producing T_H_1-type iNKT cells with augmented cytotoxicity, compared to their counterparts of DP cell origin (50). Of note, such “preferential” development of T_H_1-type cells appears to be a general attribute of unconventional T cells that are generated as a result of early TCR expression at the DN stage of thymic ontogeny (73).

A potential mechanism for the “preferential” development of T_H_1-biased iNKT cells might be related to the differentiation stage of precursor cells undergoing positive selection. In this context, it was shown that DN-stage thymocytes normally express the IL-7 receptor (IL-7R), downregulate its expression after differentiating into the DP stage, and then reexpress it as post-selection αβ T cells (74). It was reported that IL-7R determines the fate of cytotoxic effector cells via induction of Runx3, which upregulates genes associated with cytotoxic lineage cells (75). In line with this, gene expression-profiling experiments revealed that the iNKT cells of DN cell origin had elevated expression of the IL-7R and its downstream associated genes characteristic of cytotoxic cells, such as *Runx3, Gzmb*, and *Prf1*, compared to iNKT cells of DP cell origin (50).

In addition to their functional bias, NKT cells of DN cell origin had a peripheral distribution pattern different from that of NKT cells of DP cell origin. NKT cells of DN cell origin were present mainly in the liver, a result that could be explained in part by their elevated expression of genes encoding liver-homing factors. In contrast, the NKT cells of DP cell origin were present mainly in the spleen but also in mesenteric lymph nodes, lamina propria, and adipose tissues, and they had high expression of genes encoding the homing factors responsible for peripheral localization, such as CCR6, CCR7, and CCR9 (50, 76–78).

Collectively, the above results suggest that the acquisition of diverse functional characteristics by iNKT cells might be dependent on the timing of TCR expression as well as on the differentiation stage of precursor cells undergoing positive selection.

## T Cell Receptor Signal Strength

Recently, two groups reported that the TCR signal strength directs the differentiation of iNKT functional subsets (79, 80). Both studies made use of the well-characterized SKG mouse strain, which contains a hypomorphic ZAP70 allele due to a spontaneous mutation in an SH2 domain. ZAP70 (ζ-chain-associated protein kinase of 70 kDa) is a Syk family tyrosine kinase that is activated upon engagement of TCR and phosphorylates the linker of activated T cells (LAT) and the SH2 domain-containing leukocyte protein of 76 kDa (SLP-76), and thus is thought to play an essential role for T cell development (81–84). Strikingly, analyses of iNKT cells from SKG mice demonstrated that decreased TCR signaling strength leads to a predominance of NKT1 cells, whereas iNKT2 and iNKT17 cell subsets are reduced (79, 80). Based on the above results, it was proposed that higher TCR signals are necessary for the development of iNKT2 and iNKT17 cells, while iNKT1 cell development is relatively undisturbed in the context of reduced TCR signaling capacity (79, 80).

In contrast to the above, analyses of iNKT cells from the YYAA mouse strain (85), another mouse model of hypomorphic ZAP70, revealed that while the frequency of the iNKT2 subset is reduced, the proportion of iNKT17 is actually higher than that of wild-type mice and the percentage of iNKT1 cells is unchanged (80). In addition, more recently, it was shown that the iNKT1 subset is reduced in CD247^6F/6F^ mice, in which the TCR signaling capacity is reduced by ~60% as a result of phenylalanine (F) substitution of tyrosine phosphorylation sites of the six endogenous immunoreceptor tyrosine-based activation motifs (ITAMs) of CD3ζ, an obligate signal transducer of the TCR/CD3 complex (86).

Moreover, deficiency in the TCR signaling-independent transcription factor SOX4 results in specific reduction of the iNKT1 subset (86). In Sox4-deficient thymocytes, the levels of miR181, which regulates the TCR signaling threshold of DP thymocytes (87), are diminished (86). While the iNKT cell development is impaired in miR181-deficient mice (88), residual iNKT cells in these mice show increased proportions of the iNKT2 and iNKT17 subsets at the expense of reduced frequency of the iNKT1 subset (89). The above-mentioned defects in the iNKT cell development and in the proportion of iNKT subsets seen in miR181-deficient mice are normalized upon introduction of a pre-rearranged Vα14 iTCR transgene (89). Furthermore, the differentiation and functional diversification of PLZF-expressing γδNKT cells occur completely unperturbed in the absence of miR181 (90). As agonist selected T cells depend on miR181 expression, this suggests that γδNKT cells are not agonist selected. Nevertheless, these cells acquire PLZF expression and the ability to produce IFN-γ (with and without miR181), and they expand in the liver in the absence of iNKT cells. These findings argue against a sole role of agonist TCR signals to govern later functional differentiation of innate-like T cells. It was also reported that autophagy influences the iNKT functional maturation, whereby the iNKT1 cell subset is mostly affected via regulation of the cell cycle and survival processes (34, 91, 92). Additionally, let-7 and miR-17 contribute to iNKT subset development via post-transcriptional regulation of PLZF or TGF-βR II expression in post-selection iNKT cells, respectively (93, 94).

Collectively, the current literature does not provide a clear consensus interpretation on how differential TCR signaling strength affects iNKT functional maturation into distinct subsets. Further complicating the matter, it is possible that the kinetics of acquiring TCR signals over time might be as important as the avidity or quantity of individual TCR signaling events *per se* (95). Sub-lineage choices might occur based on whether TCR signaling persists or ceases as the case of conventional CD4 T or CD8 T cell choice proposed by the kinetic signaling model (96). It is also possible that positive selection and sub-lineage choices are sequential but not simultaneous events. Finally, other undefined TCR-independent factors provided by the microenvironment might affect the differentiation of iNKT functional subsets, as it was reported that iNKT1, iNKT2, and iNKT17 subsets develop, albeit with subtle variations, in mouse models with the monoclonal iNKT TCR specificity (22, 97).

## Concluding Remarks

Despite tremendous progress in the field, a number of important questions regarding the development of iNKT cell subsets remain unanswered. First, it is not completely understood why strong agonist signaling, which normally results with the clonal deletion in conventional T cells, culminates in the positive selection of the iNKT cell lineage. Second, how stable are these functional subsets and can they interconvert? In this context, it remains unknown what iNKT cell subsets are the precursors of iNKT_FH_ and iNKT10 cells. Third, what are the factors that dictate homing and maintenance of iNKT cell subsets to different tissue sites? As currently there is no consensus view on the precise mechanisms driving the development of the functionally distinct iNKT sub-lineages, it is tempting to hypothesize that multiple mutually non-exclusive mechanisms might exist. A better understanding of functional differentiation mechanisms of the iNKT cell lineage could contribute in developing optimized strategies intended to exploit the unique features of iNKT cells for the benefit of patients.

## Author Contributions

ND wrote the first draft. ND, SB, and MS-S edited the manuscript.

### Conflict of Interest Statement

The authors declare that the research was conducted in the absence of any commercial or financial relationships that could be construed as a potential conflict of interest.
